# Evidence for miniscalpel-needle/needle knife in the management of chronic pain related conditions: A protocol for systematic review and meta-analysis

**DOI:** 10.1097/MD.0000000000016474

**Published:** 2019-07-12

**Authors:** Di Zhang, Ying Cheng, Guixing Xu, Zihan Yin, Jiao Chen, Fanrong Liang

**Affiliations:** Chengdu University of Traditional Chinese Medicine, Chengdu, China.

**Keywords:** chronic pain, miniscalpel-needle, myofascial syndrome, needle-knife, soft tissue, systematic review, trigger point

## Abstract

**Objective:**

The aim of this systematic review with meta-analysis is to determine the effect of miniscalpel-needle/needle-knife in treatment of chronic pain symptoms.

**Methods:**

The following electronic databases will be searched by 2 independent reviewers: PubMed, Cochrane Library, EMBASE, Springer, China National Knowledge Infrastructure (CNKI), Wanfang, and Chinese Biomedical Literature Database (CBM). All randomized controlled trials on miniscalpel-needle/need-knife for chronic pain published in electronic databases from inception to August 1, 2019 with language restricted in Chinses and English will be included in the study.

Methodologic quality is assessed by 2 blinded reviewers independently screen and score the articles using the Physiotherapy Evidence Database (PEDro) scale and the Cochrane Collaboration risk of bias tool. A meta-analysis was performed when there is sufficient clinical homogeneity in at least 2 studies. The Grading of Recommendations Assessment, Development and Evaluation approach is used to rate the body of evidence in each meta-analysis. When the quantitive evaluation is not available, a qualitative description of the results of single study is provided.

**Results:**

A high-quality synthesis of current evidence of miniscapel-needle/needle-knife treating chronic pain will be illustrated using subjective reports and objective measures of performance. The primary outcomes consisted of pain intensity improvement rate clinically meaningful improvements in pain and disability are also noted. Secondary outcomes involve the short form of McGill Pain Questionnaire score (SF-MPQ) and the side effects.

**Conclusion:**

This protocol will present the evidence of whether miniscalpel-needle/needle-knife is an effective intervention for chronic pain.

**PROSPERO registration number:**

CRD42019129076

## 1 Introduction

Miniscalpel-needle (MN)/needle-knife (NK) or needle knife is a Traditional Chinese Medicine intervention widely used to treat chronic pain conditions,^[1–5]^ particularly for myofascial and associated dysfunction all over the world. Myofascial pain syndrome is characterized by the presence of 1 or more trigger points (TrPs) located in skeletal muscle. Trigger points^[6]^ are palpable, localized areas of hyperalgesia muscle tissue typically located in a tough band of fibers, which is called pain acupoint in Chinese acupuncture. Trigger points can lead to local muscle contractures due to an excessive release of acetylcholine, increased activation of nicotinic receptors and inhibition of acetylcholinesterase at the motor endplate, causing loss of range of motion (ROM), weakness and painful contractions.^[7–9]^ Several hypotheses exist to explain the physiological mechanism behind sign and symptom reduction with MN/NK.^[10–14]^ It has been suggested that MN/NK hyper-stimulates the pain-generating area and thereby normalizes the local sensory inputs.^[15,16]^ Another hypothesis suggests that MN/NK causes natural opioid-mediated pain suppression by stimulating local alpha–delta nerve fibers.^[17]^

### 1.1 Description of the intervention

MN/NK or Needle knife is a kind of metal needle which resembles both needle and knife in shape. It was developed on the basis of 9 needles in ancient times and combined with surgical scalpel. Its diameter ranges from 0.4 mm to 1 mm and its length ranges from 6 to 15 cm.

MN/NK is administered by inserting a thin, solid needle with broad needle point directly into the palpable trigger point or pathological sensitive points of nerve conduction, ganglions, joints, muscles, facia, tendons, ligaments, and so on. The length and the shape of needle point are varied and flexible, depending on individual acupuncturist's personal like and expertise.^[18]^ Normally, the MN/NK needle is thicker than clinical filiform needle, and the needle point is broader in order to manipulate within the tissue to elicit a localized twitch response, to stimulate or block nerve conduction, to cut, release fascia or muscle impingement points and peel off of old harmful tissue fibers^[19]^ so as to relieve pain and dispel disease. The treatment is simple, not limited by any environment and conditions. And the wound is small and needn’t be sutured. It has little tissue damage and no obvious pain and fear for patients.

Risks of needle insertion are relatively higher than filiform needle. And the most common adverse events with regular MN/NK include bruising, bleeding and pain, occurring at a rate of 20%.^[20]^ These events are classified as mild because they are short-term and do not require further medical treatment. Additional risks associated with MN/NK may include hematoma, infection, pneumothorax, and nerve lesions.^[21–23]^ Several recent systematic reviews have shown positive results with MN/NK; however, these reviews reported limited conclusions due to low-quality studies.

The purpose of this systematic review is to evaluate high-quality randomized controlled trials (RCTs) to determine the immediate and long-term effectiveness of MN/NK as a treatment for chronic pain related to trigger points located in multiple body regions.

## 2 Methods

### 2.1 Selection criteria

#### 2.1.1 Types of studies

High-quality RCT's investigating MN/NK treatment are included as shown in Table 1.

**Table 1:**
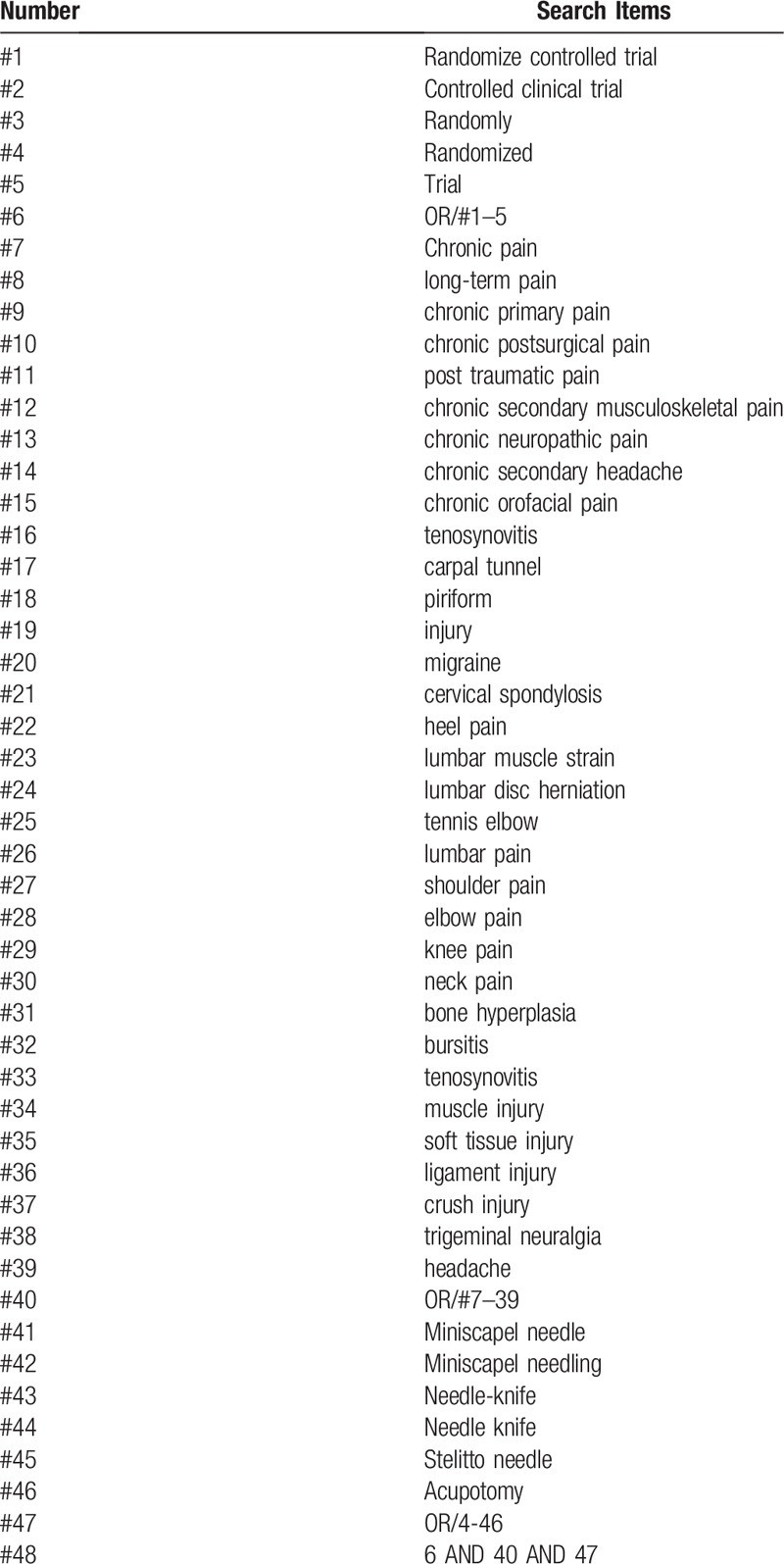
illustrates the search strategies applied to this review.

#### 2.1.2 Types of participants

We will identify RCTs with a control group with non-specific chronic pain (>12 weeks duration), which contained drug, no treatment, placebo, diet and exercise therapy; other types of acupuncture including fine needles, fire needling, electronic needling, ear auricular pressure treatment, acupoint pressure, and so forth are excluded. Trials with mixed subacute (>6 weeks duration) and chronic pain populations will also be eligible for the Individual Participant Data (IPD) meta-analysis as it will be possible to extract information on chronic participants.

Chronic pain participants must conform to the International Classification of Diseases (ICD 11) released by WHO in 2018, with no limitation of age. Type of chronic pain conditions of this study including chronic primary pain, chronic postsurgical or post-traumatic pain, chronic secondary musculoskeletal pain, chronic neuropathic pain, chronic secondary headache or orofacial pain, and other chronic specified or unspecified pain. The definitions of chronic pain symptoms are included. Patients with acute medical conditions or pregnancy are excluded.

#### 2.1.3 Types of intervention

The review comprises clinical trials with the treatment of MN/NK. We will study the types of acupuncture including needle knife, mini-scalpel needle, acupotomy, acupotome, and stiletto needle. We also included trials that compared MN/NK plus another active treatment versus other active treatment alone.

#### 2.1.4 Types of comparator(s)/control

The following control groups will be considered:

1. sham devices
2. pain killer
3. placebo
4. conventional drugs
5. other clinical control group
6. MN/NK in addition to active treatment versus active treatment alone.

Studies that only compare different forms of acupuncture are excluded.

#### 2.1.5 Types of outcome measures

The primary outcomes consisted of pain intensity improvement rate: average visual analog score (VAS) 1 to 10 and pain frequency in the short term and upon long term follow-up for each subject; clinically meaningful improvements in pain and disability are also noted.

Secondary outcomes involve the short form of McGill Pain Questionnaire score (SF-MPQ) and the side effects, such as pneumothorax, bleeding, serious discomfort, subcutaneous nodules, and infection.

### 2.2 Data source

A computerized search of electronic databases of PubMed, Cochrane Library, EMBASE, Springer, China National Knowledge Infrastructure (CNKI), Wanfang, and Chinese Biomedical Literature Database (CBM) will be conducted. Unpublished articles will be manually searched in Google scholar.

### 2.3 Search strategy

All randomized controlled trials on miniscalpel/need-knife for chronic pain published in electronic databases from inception to January 1, 2019 with language restricted in Chinses and English will be included in the study. The Medical Subject Headings (MeSH), text words, and word variants for “chronic pain” Chronic pain”, “long-term pain”, “chronic primary pain”, “chronic postsurgical pain”, “post traumatic pain”, “chronic secondary musculoskeletal pain”, “chronic neuropathic pain”, “chronic secondary headache”, “chronic orofacial pain”, “tenosynovitis”, “carpal tunnel”, “piriform”, “injury”, “migraine”, “cervical spondylosis”, “heel pain”, “lumbar muscle strain”, “lumbar disc herniation”, “tennis elbow”, “lumbar pain”, “shoulder pain”, “elbow pain”, “knee pain”, “neck pain”, “bone hyperplasia”, “bursitis”, “tenosynovitis”, “muscle injury”, “soft tissue injury”, “ligament injury”, “crush injury”, “trigeminal neuralgia”, “headache” and “miniscalpel needling” or “miniscalpel needle” or “scalpel needle” or “scalpel needling” or “acupotomy” or “needle-knife” or acupotome” or “acupotomology” or “Stiletto needle” are used and combined in the searches. Exclusion criteria: duplicates, non-human participants, non-English or Chinese language, exclusive focus on acupuncture or medicinal injections. We will conduct citation searches and contact content experts for additional trials. Hand searches of key musculoskeletal journals are captured in the Cochrane Central Register searches. Manual searching is conducted by searching reference lists in relevant articles and unpublished research.

### 2.4 Data collection and analysis

#### 2.4.1 Screen of studies

**Figure 1:**
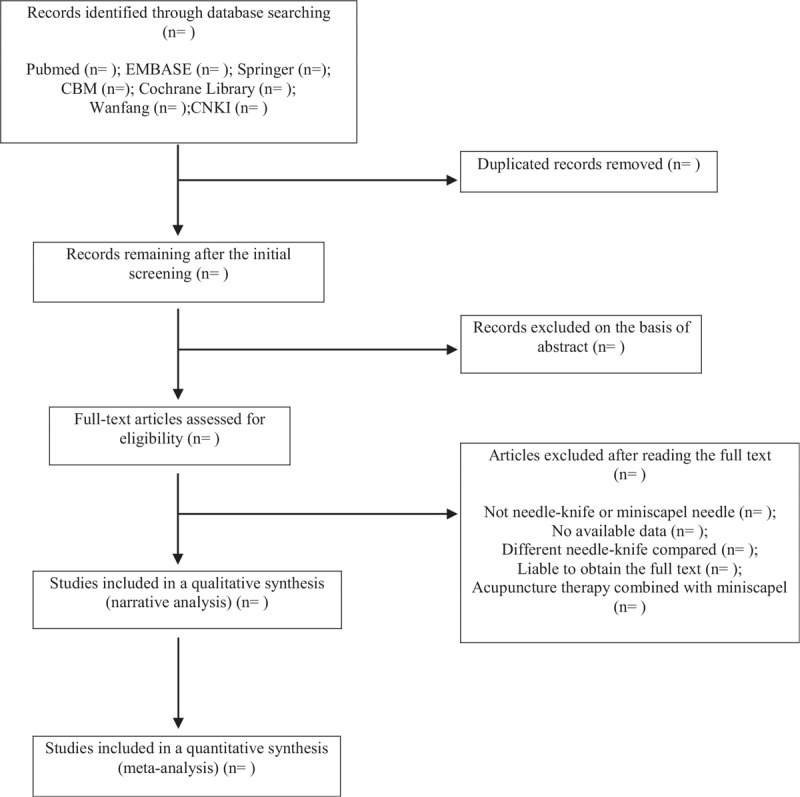
illustrates the flow diagram of studies identified.

Two (YZH and XGX) authors will independently scan the titles and abstracts. The studies that satisfied the inclusion and exclusion criteria are retrieved for full-text assessment. For cross-over trials, the summary data are used as if they have been derived from parallel trials. For trials with more than 2 intervention groups, the experimental group is compared with the control group by combining the data of all relevant control groups. Details of the selection procedure for studies are shown in a PRISMA-P flow chart (Fig. 1).

Articles are excluded based on the following criteria: duplicates, non-human studies, and non-English/Chinese language studies. All articles meeting search terms are scrutinized to ensure that MN/NK is executed as either an intervention. The animal mechanism studies, case reports, self-pre- and post-control, or non-RCTs are excluded. Studies to compare the effect of different acupuncture therapies will be excluded.

#### 2.4.2 Data extraction

Data are extracted by 2 independent reviewers from each included article using a standard form (based on the Cochrane data extraction template) including the following information: patient characteristics, similarity between groups, intervention and control information, blinding, outcome measures, times to outcomes, statistics used and results with supporting data, and the Standards for Reporting Interventions in Controlled Trials of Acupuncture (STRICTA) checklist. The remaining discrepancies in data extraction are resolved after the discussion between the 2 reviewers. A third reviewer (LFR) will adjudicate when necessary. The results regarding the outcome measures are extracted in the form of mean and standard deviation data.

#### 2.4.3 Quality of study

The (PEDro) scoring system was used to evaluate research quality. The PEDro score is based on 11 criteria, 10 of which contribute to the overall score of an article. An article may obtain a maximum of 10 points on the PEDro score, reflecting internal validity and sound statistical analysis. Randomized controlled trials with a PEDro score of 6 or greater are included in this systematic review.

#### 2.4.4 Assessment of risk of bias

Risk of bias is used to evaluate the quality of study with the Cochrane Collaboration's risk-of-bias assessment method and complete the STRICTA checklist for the included studies. The decision of risk is made by 2 reviewers (CY and YZH). If inconsistent results appear, the final decisions will be made by the third author (LFR). For missing or ambiguous data, we will try to contact the author as possible, and for duplicate publication, we only select the original. When the individual researchers scored articles differently, score discrepancies are resolved by reviewing articles together as a group and evaluating each scored item together. In this way, consensus will be reached among all researchers for every RCT without disagreement. The Preferred Reporting Items for Systematic Reviews and Meta-Analyses (PRISMA) checklist is utilized to ensure clear, thorough reporting in this review.

#### 2.4.5 Unit of analysis issues

The analysis will focus on patients in randomized studies. If more than 1 objective is used, we will conduct separate multiple meta-analyses for each treatment objective. If multiple non-MN/NK control groups are included, pooled analyses of the control groups against the intervention group will be used.

#### 2.4.6 Management of missing data

There are missing or incomplete data for the primary results; we will contact the corresponding authors for the missing data. If the missing data cannot be obtained, it will be included for narrative analysis.

### 2.5 Data synthesis

#### 2.5.1 Narrative analysis

We may conduct narrative synthesis if meta-analysis is not appropriate (e.g., incidence of adverse events of MN/NK). We will describe and compare study-level characteristics and aggregate data from studies participating in the IPD analysis with those from studies that are eligible but do not supply data to the collaborative; we will identify if the IPD studies available are a representative (unbiased) statement of the full set of existing studies.

#### 2.5.2 Meta-analysis

RevMan V.5.1 will be employed for original data analysis when meta-analysis is possible. The mean differences (MD) with a 95% confidence interval (CI) will be used to assess continuous outcomes, while the risk ratio (RR) with a 95% CI will be used for dichotomous data.

#### 2.5.3 Assessment of heterogeneity

The heterogeneity test will be performed on the included studies by using I^2^ test to estimate the included studies. Review Manager (version 5.1, the Nordic Cochrane Centre, Copenhagen, Denmark) is applied to assess curative effect and publication bias. Forest plot is used to illustrate the relative strength of curative effect. Meanwhile, the funnel plot will picture the publication bias visually as the number of trials is more than 10. If significant heterogeneity is detected, a random-effects model will be used.

We define that: 0% -30% is mild heterogeneity, 30% to 60% is moderate heterogeneity; 60% to 100% is large heterogeneity. When I^2^ ≤30%, the RR and MD will be calculated by a fixed-effects model. If I^2^ >30%, a random-effects model will be used to synthesize the data. Subgroup analysis or meta-regression will be conducted to explore the causes of heterogeneity including clinical or methodological reasons. Sensitive analysis will be conducted to test the stability of this synthesis.

#### 2.5.4 Assessment of publication biases

Funnel plots are used to assess reporting biases. If funnel plot asymmetry is detected, the reasons will be analyzed.

#### 2.5.5 Subgroup analysis

A subgroup analysis will be performed according to control intervention, intervention frequency, and different outcomes.

#### 2.5.6 Sensitivity analysis

A sensitivity analysis will be performed according to the following criteria: sample size, heterogeneity qualities, and statistical model (random-effects or fixed-effects model).

## 3 Discussion

MN/KN is a very thin needle with broader point that your therapist pushes through the skin to stimulate the trigger points or cut the abnormal tissues, muscles, or connecting tissues. MN/KN may release the tight muscle bands and decrease pain. The resulting larger sample size and consistent presentation of data will allow additional analyses to explore patient-level heterogeneity in treatment outcomes and prognosis of chronic pain. The results of this review indicate that multiple protocol-related variables may influence MN/NK outcomes. Individual study designs included here differently manage potential key variables, such as pain chronicity, needling technique, number of treatment sessions, presence of adjunct interventions, outcomes measured and time to outcome measurement.

A wide variety of acupuncture protocols are used in the RCT's reviewed, which complicates comparison of study outcomes and possibly jeopardizes the external validity of this review. Therefore, multiple outcome measures should be explored per study, with measures over time, in order to better understand the potential benefits of MN/NK and guide clinical decision-making.

Results are limited by the quality and sample size of English/Chinese articles. Access to new, unpublished data may not available by the time this review is submitted; therefore, the results presented here may be influenced by publication bias.

## Author contributions

**Funding acquisition:** Fanrong Liang.

**Investigation:** Guixing Xu.

**Methodology:** Zihan Yin.

**Supervision:** Jiao Chen, Fanrong Liang.

**Writing – original draft:** Di Zhang, Ying Cheng.

**Writing – review and editing:** Di Zhang, Ying Cheng.
